# Value-Based Care in the Worldwide Battle Against Cancer

**DOI:** 10.7759/cureus.1039

**Published:** 2017-02-17

**Authors:** Niloufer J Johansen, Christobel M Saunders

**Affiliations:** 1 Oncology Clinical Trials Unit, St John of God Subiaco Hospital; 2 School of Surgery, University of Western Australia

**Keywords:** value based health care, cancer care, data-capture, global, value-based healthcare

## Abstract

Globally, an increasing and aging population is contributing to the prevalence of cancer. To be effective, cancer care needs to involve the coordination of multidisciplinary specialties, and also needs to be affordable, accessible, and capable of producing optimal patient outcomes. Porter and Teisberg (2006) have postulated that shifting current healthcare strategies from volume-based to patient-centric care redirects economic competition to providing treatments which promote the best patient outcomes while driving down costs. Therefore, the value in value-based healthcare (VBH) is defined as patient outcome per currency spent on providing care. Based on the experiences of healthcare organizations currently transitioning to the value-based system, this review details actionable guidelines to transition current cancer care practices to the value-based system in four main steps: by defining universal clinical and patient-reported measures, creating cancer-specific units that provide the full care cycle, establishing a data capture model to routinely determine the value of the care delivered, and continually improving treatment strategies through research. As healthcare providers in more developed countries move to value-based care, those located in less developed countries should also be assisted in their transition to relieve the cancer burden globally.

## Introduction and background

Cancer can affect any individual regardless of his or her race, socioeconomic status, or geographical location. The Global Burden of Disease Study (GBD) 2015 reported that between 1990 and 2015, global mortality rates due to cancer have risen by 17%, making it the second largest contributor to deaths by a non-communicable disease [1]. Furthermore, between 2005 and 2015, both population growth and an aging population contributed to the 33% increase in the number of cancer cases worldwide [2]. These estimates may rise further as more data from low-income and middle-income countries are incorporated [1]. Encouragingly, the GBD 2015 study found a reduction in the age standardized death rates for most cancers, attributed to a reduction in risk factors and improvements in selected healthcare systems providing early diagnoses and targeted therapies. While the overall goal of health systems is to extend life and promote healthy living, it is predicted, as life expectancy increases, mortality rates due to non-communicable diseases will increase, placing greater pressure on existing health service providers [1]. 

###  Challenges facing cancer care in the current climate

**Figure 1:**
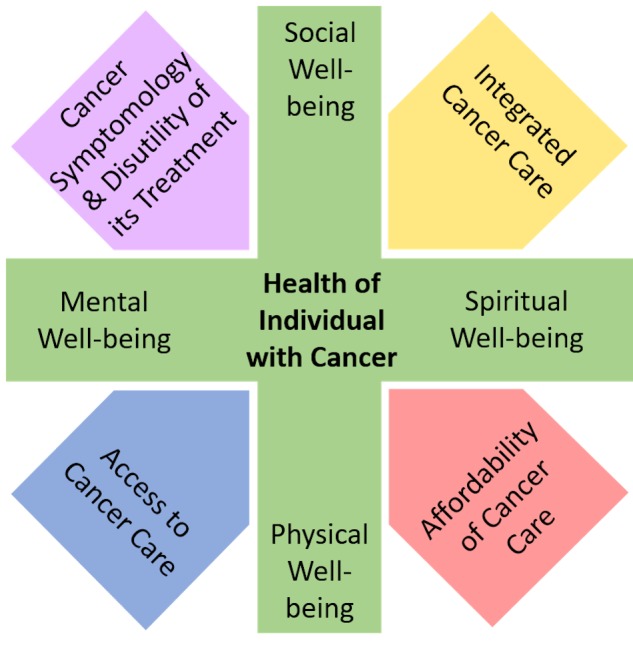
Factors contributing to the health of an individual diagnosed with cancer.

The World Health Organization defines health as “a state of complete physical, mental and social well-being and not merely the absence of disease or infirmity” [3]. While this definition attempts to emphasize a more holistic approach to understanding an individual’s state of health, it is unlikely that a person will be content in all these attributes for a considerable length of time. Figure 1 summarizes the factors relating to the individual (e.g., physical, social, mental, and spiritual) and cancer care (e.g., cancer symptomology and disutility of its treatment, integratedness, and affordability and access) that can determine the overall health of a person diagnosed with cancer. Cancer requires care from many specialties from diagnosis to possible long-term survival [4]. While research has yielded treatments that have substantially improved prognosis for some cancers, health is still partly determined by the affordability and accessibility of treatments both in lesser developed and more developed nations [1-2, 5]. Screening has reduced mortality rates in some cancers [6], contributing to a growing population of cancer survivors; and thus, focus of clinical management has shifted towards long-term survivorship [7]. Furthermore, as more patients survive their cancers, more research is required to understand recurrence, the risk of developing other cancers and/or medical conditions, and the integration of "survivors" into society [7].  

Globally, healthcare systems are plagued by a combination of rising costs, reduced access to good medical care, and a lack of transparency and coordination in assisting the delivery of effective treatment. Porter and Teisberg argue that competition at various levels in healthcare (e.g., hospital-based stakeholders, insurance providers, financial payers, and suppliers) to perpetuate effective medical care had failed [8]. The value was not based on enhancing long-term patient outcomes, but on short-term cost-saving cycles which focused on the clinical absence of disease [8]. They and others argue that healthcare is seen as a commodity where all health services are the same, and all patients, regardless of disease type, have the same needs [8-9]. The end result has been the duplication of services but a reduction in the choice and quality of treatment [8]. Lack of standardization in measuring enhanced long-term patient outcomes has contributed to limited transparency in comparative treatment performance and the spread of non-evidence-based treatment-related information and practices [8]. 

###  Redefining value in cancer care

Value-based healthcare (VBH) focuses on improving patient health outcomes while reducing the overall cost of healthcare [8]. The value of VBH depends upon the best patient outcome after treatment for a given disease. By redefining value in healthcare, the aim is to change the nature of competition to drive improvements in the quality and cost of treatments and/or management processes that improve long-term patient health. By focusing on patient outcomes, the focus is redirected towards treating the medical condition which determines the medical needs of a patient. Table 1 summarizes selected Porter and Teisberg’s principles of value-based competition which relate to cancer care [8].  

**Table 1:** Selected Porter and Teisberg’s principles of value-based competition relevant to cancer care.

Selected Principles of Value-Based Competition
Refocus on the value for patients rather than lowering costs consistently at all levels within the healthcare institution
Competition is driven by results, i.e., favor processes which demonstrate improved value of the care delivered
Reduce cost of high-quality care
Competition should expand from local areas to regional and national settings
Provide transparency and accessibility of value-based results from all participants in value-based care
Reward and endorse innovations that better the value of care provided for a medical condition

## Review

In order to transition current cancer care into a value-based system that encompasses these principles, the following need to occur:

1.     For every type of cancer, a longitudinal dataset needs to be defined which measures clinical and patient-reported outcome measures (PROMs) that can be applied universally.

2.     Specialty-oriented departments need to be transitioned into multidisciplinary cancer-specific units which provide the full care cycle. 

3.     A data-capture-and-analyze model needs to be implemented to routinely determine the value of the delivered cancer care.

4.      Cancer care should be continually improved through research.

### Define clinical and patient-reported outcome measures

Evaluating the effectiveness of any treatment regime for a disease requires measurement of patient outcomes, for patients treated with both curative and palliative intent, in addition to parameters of clinical improvement. The International Consortium for Health Outcomes Measurement (ICHOM) has developed a number of standardized datasets to measure both clinical and patient-oriented value-based health outcomes in cancers of the breast [10], bowel [11], lung [12], and prostate [13-14]. While numerous datasets exist, not all questions are relevant to each medical condition. Furthermore, it is important to establish a universally-applicable dataset which incorporates new measurement initiatives for ease of implementation [10-14]. For each of the currently developed cancer datasets, ICHOM established a global multidisciplinary working group of experts and patient advocates who identified relevant and valid outcome measures and developed disease-specific indicators of cancer progression and survival, disutility of care, and/or complications and PROMs most relevant to patients. Annual renewal [13-14] is recommended. By 2017, ICHOM aims to complete the development of datasets for 50% of medical conditions contributing to the total measured global disease burden [11], including for a number of other cancers. 

### Create cancer-specific multidisciplinary units providing the full care cycle

The engagement of hospital- and patient-based stakeholders is imperative to transition specialty-oriented departments into cancer-specific multidisciplinary practice units which provide the full care cycle [15-16]. Restructuring existing facilities requires a significant investment in terms of time and resources which can only occur through visionary leadership at the management level [16]. Realigning services with patient needs for a specified medical condition is fundamental [8]. It is therefore important that the full care cycle is determined first, followed by the development of individual cancer-specific multidisciplinary practice units. Current payment systems need to transition from a fee-for-service to a payment program that streamlines treatment-associated costs and provides transparency when providing quotes at the onset of care [8, 17]. To allow for the financial transition from fee-for-service to a bundled payment program (as being explored in the current Australian review of Medicare funding; personal communication) the cancer-specific multidisciplinary practice unit would need to define the time and costs required to complete a full care cycle [15].

Defining the full care cycle involves itemizing the services, treatments, and timeframe required to address a medical condition from diagnosis to assessment of treatment outcomes [8, 15]. Defining the full care cycle therefore determines the structure of the multi-disciplinary team necessary for a viable cancer care practice unit [16]. Centralizing the multi-disciplinary team to one care location allows for a more efficient and unified approach to multi-disciplinary care and fosters a culture of education, improvement, and innovation in care delivery [16], although other successful hub and spoke models certainly exist.

In each cancer unit, multi-disciplinary teams gather regularly to discuss and define management plans for all cancer patients [16], resulting in improved patient outcomes [4]. This offers a more unified approach to interpreting clinical results, treatment decision-making, and cross-disciplinary communication in the care provided [4, 18]. Conducting multidisciplinary meetings (MDMs) requires investment in time and finance [18-19]. With the establishment of medical condition-specific datasets, it is possible to study the impact of MDMs on patient outcomes and prospectively measure their efficiency in clinical care processes in terms of meeting frequency, how the meeting is conducted, and the number of cross-referrals within and between institutions.

It is currently unknown which payment scheme will yield the best value. Because time-driven, activity-based costing (including professional staff, direct costs, and overheads) is rare in healthcare, M D Anderson Cancer Center has trialed a bundled payment program which separated costing into three episodes of care at pretreatment, treatment, and post-treatment phases [15]. This allowed for a better understanding of where the costs were allocated during the course of care and differences in pricing due to the severity of the medical condition [15]. The implementation of a bundled payment program can minimize errors in calculating costs and streamline administrative processes, thereby creating a more efficient, reliable payment system. It is likely that one value-based payment model will not fit all healthcare scenarios and, therefore, it is important that other schemes, e.g., accountable care organizations and pay for performance under the Affordable Care Act in the USA, are trialed [20-21].

### Establish a data-capture-and-analyze model to routinely determine the value of the delivered cancer care

**Figure 2:**
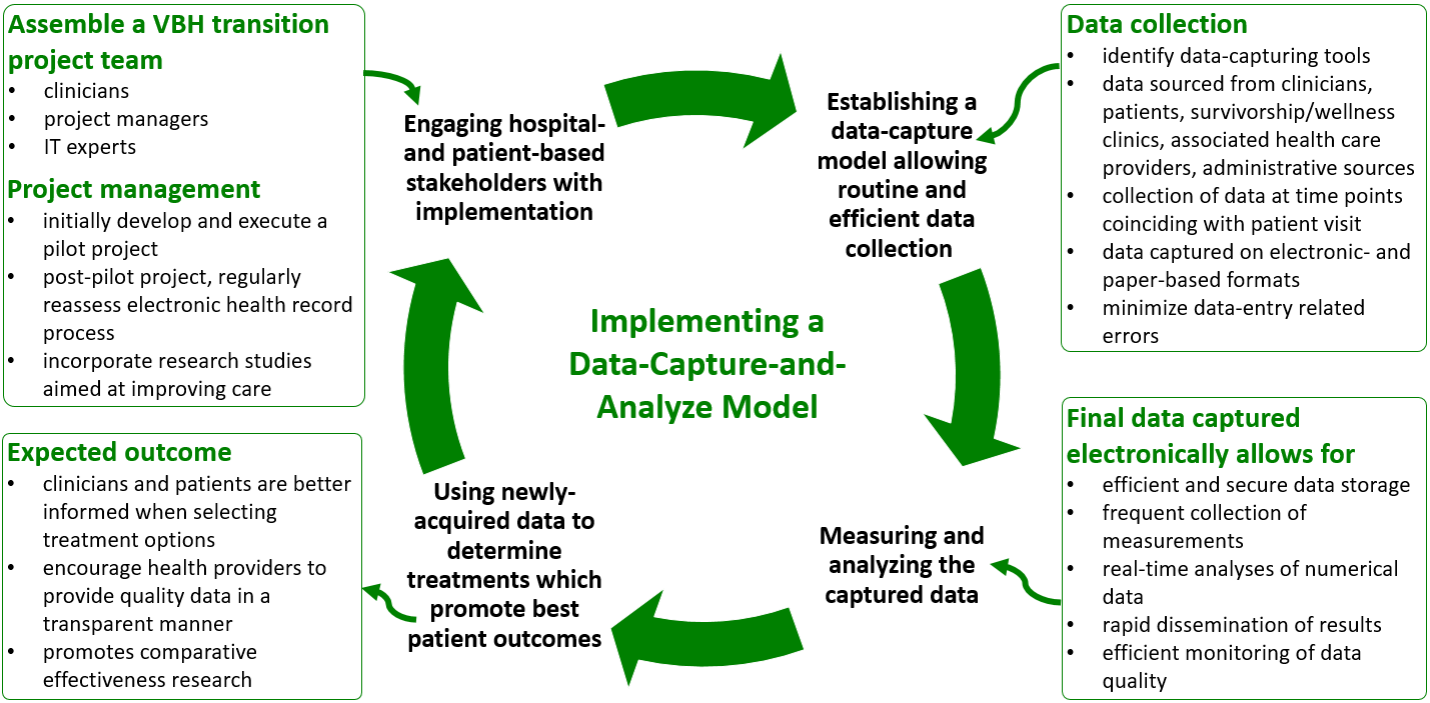
Establishing and implementing a data-capture-and-analyze model to assess the value of cancer care

Establishing a data capture model is important in routinely and efficiently assessing the value of the cancer care received by patients (Figure 2). This involves extracting data from clinical, patient-based, and administrative source data. PROMs and clinical data, weighted by the cost of treatment, can then determine the value of care provided. To the best of our knowledge, three providers of cancer care have published their experiences in successfully implementing a dataset measuring patient outcomes in conjunction with clinical data and treatment-related accounting measures: Head and Neck Cancer [15] and Breast Care [22] Centers at the M D Anderson and, more recently, the Cleft Department at Sophia Children’s Hospital, Erasmus Medical Center, Netherlands [23]. Only the Cleft Department at Erasmus Medical Center has reported the implementation of the full ICHOM dataset, whereas reports of implementation at MD Anderson refer to datasets developed at the institution themselves prior the development of ICHOM-based datasets [11]. Based on their experiences, considerations on strategies employed to successfully implement a standardized dataset in cancer care are summarized in Figure 2.

Importance of IT in Dataset Implementation

Critical to implementing the dataset is the electronic capture of data which can then be analyzed using an electronic heath record (EHR) system or electronic medical practice. There is evidence to suggest that patient outcomes and clinical productivity have improved using EHR systems, in comparison to paper-based methods, by improving data completion and reducing the potential for physical loss of data and transcription errors and minimizing data entry [24]. Current EHR systems, however, have been geared towards capturing data efficiently but lack the ability to easily export this data into an analyzable format [15]. An EHR system requires flexibility in extracting data from multiple sources (e.g., PROMs, clinical, accounting, and administrative) in a manner that avoids duplication in data entry [23]. Importantly, this IT platform needs to allow for the captured data to be analyzable and reportable on an ongoing basis for quality control and research purposes. Due consideration should be given to how patient health data is stored and used by devising consent forms which allow the patient to use their data in different healthcare institutions but which can also be used in non-commercial research settings [25]. Finally, the EHR needs to be adaptable in addressing future needs. Therefore, establishing and maintaining such an EHR would require continuing IT support [23].   

Assemble VBH Transition Project Team

The first step in implementation is to form a project team consisting primarily of individuals who champion the core principles of value-based health [11, 22-23]. Such individuals would include clinicians, project managers, IT experts, and importantly, the consumers themselves [23]. Two reported projects have demonstrated the ability to implement a patient outcomes based dataset in a pilot setting within a one-year timeframe [22-23]. 

Develop and execute a pilot project

Piloting such a system in an individual institute is a good first measure to ensure success in implementation [23]. It defines when, where, and how the data are obtained during the clinical care cycle and determines access levels to the information accessed by patients, e.g., allow patient to fill in/review PROMs-based information but restrict access to all other health-related information located on the hospital server. The sources for the remaining data required to complete the dataset are also identified, as are the data-collection pathways. Electronic questionnaires can be developed within the IT platform, and staff can be educated and trained in implementing this in practice [23].

Patient reported outcomes can be collected electronically via a patient portal accessible within the hospital (e.g., at M D Anderson Breast Cancer Care) or via online surveys accessible via hyperlinks sent by email (e.g., at Cleft Department, Erasmus Medical Center) [16, 22-23]. To encourage data collection compliance of PROMs, patients should be afforded more than a single opportunity to complete questions [23]. Patients and clinicians can be recruited in conjunction with routine clinical visits [11]. The pilot project allows a unique opportunity to establish how patients interpret the language of the questionnaires. For instance, at the Breast Care Center at M D Anderson, participants preferred language such as “what my life will be like” or “medical results” over the word “outcomes” [22]. 

Systematic roll-out of EHR

The EHR should be continually reviewed to assess how it can be improved in terms of implementing new data fields, sourcing data, how data is analyzed and presented (e.g., provision of a dashboard to visualize care-related metrics), and the user interface being made more intuitive and user-friendly [23]. An important consideration is to ensure that rollout and continued adaptation is done incrementally so as to minimize disruption of routine clinical care processes [23]. Furthermore, routinely publishing reports accessible to the public allows for monitoring the efficiency and effectiveness of current treatment strategies and ensures that patients receive the best care for their respective medical condition(s) [8]. 

### Continually improve cancer care through research

Research is vital for improving current treatments by addressing both survival and quality of life, as well as informing socioeconomic and health-related policies and funding allocation by governing bodies. The involvement of clinical trials in a value-based cancer care setting allows for the continual assessment of current and new treatment strategies within a "real world setting" [26]. This involves the seamless integration of experimental strategies concurrently with standard treatment processes and care resulting in the best PROMs either remaining or being rapidly adopted [1, 8, 27]. For instance, the "control" group would be patients receiving standard care which can then be compared to those receiving either existing or new therapies [26-27]. This affords the patient access to potentially life-saving treatments that would not have otherwise been available. The Community Clinical Oncology Program (CCOP), a collaborative initiative specialized in facilitating phase III clinical trials amongst healthcare institutions in the US, demonstrated the importance of incorporating research within the institutional level [28]. The sustainability of the CCOP in an adverse economic climate has been challenging primarily due to a limited understanding of administrative processes and the requirements involved in coordinating multi-site trials [28]. Implementing medical condition-specific datasets would allow for the comparison of treatment results within and between geographical locations [8]. An EHR would allow for the efficient incorporation of modules which collect and analyze trial-related data in addition to those for standard care. Furthermore, the EHR would also allow for the rapid dissemination of results. 

### Post-implementation experiences

Evaluating the impact of transitioning into a VBH model is in its early days. Pilot implementations have suggested a positive change in the care cycle whereby everyone involved was more prepared for each clinical visit [23]. For instance, the active involvement of patients in reporting their health outcomes encouraged them to discuss health-related issues which mattered most to them. Clinicians reported that clinic visits were more structured and focused, as they had a better understanding of the patient's views regarding their treatment [23]. 

Since the implementation of EHR requires a substantial initial investment, it is not uncommon to experience a short-term decrease in net income for the care center. The M D Anderson Cancer Center, after starting the implementation of their EHR in March 2016, reported a 76.9% decrease in their adjusted income (i.e., revenue remaining after accounting for operating expense from total operating revenues), which was “primarily attributable to” this in the ten months ending in June 2016, largely due to increased payroll costs, which they anticipate will be overcome within one year [29]. It is important to note that this report is indicative of the short-term financial investment. A financial analysis in the longer term would determine if this will achieve superior patient outcomes per amount spent. It is likely that greater financial transparency from care centers will allow for driving healthy competition in improving the patient outcomes per amount spent in the full cancer care cycle (i.e., reduced cost for better care) [8].

###  Implementation in lesser developed nations

The GBD Cancer Collaboration has indicated that the rapid increase in global life expectancy was counterbalanced by unchanged or increasing rates of deaths due to diseases such as cancer [1, 5]. It is therefore important to address how value-based cancer care can be implemented to reduce cancer burden. The cancer care offered to patients in the lowest income bracket is minimal due to limited affordability and accessibility [5]. Furthermore, this is compounded by hospitals primarily specializing in treating low burden conditions resulting in minimal to no care provided to patients diagnosed with cancer [5]. 

In less developed countries, experienced healthcare providers caring for high burden medical conditions are most likely to have mechanisms in place to provide culturally sensitive care. Knowledge transfer from medical condition-specific experts with experience in value-based care is vital for these transition providers. Finally, it is important to establish simplified routes for collecting, storing, and analyzing data. A coordinated and collaborative approach in moving current heathcare systems into the new value-base care system would ensure better patient outcomes globally and further improve health outcomes linked to lower income countries.

## Conclusions

While the shift to value-based care is in its early days, it comes at a time when there is desperate need to find alternative methods of providing cost-effective care which aims to provide patients with the best possible treatment outcome. Increasing population and life expectancy predict higher incidences of cancer diagnosis and death. Although the principles of value-based care hold the promise of bettering cancer care, a wider evaluation of this paradigm remains to be undertaken. It is therefore imperative we learn from the experiences of healthcare organizations currently transitioning into VBH. Only if the early-adopters of VBH demonstrate superior patient outcomes at affordable means can subsequent adoption by other healthcare providers be encouraged so as to minimize the global cancer burden.
